# Comparative Analysis of Common Strength Criteria of Soil Materials

**DOI:** 10.3390/ma14154302

**Published:** 2021-07-31

**Authors:** Ping Xu, Zhijun Sun, Shengjun Shao, Lingyun Fang

**Affiliations:** 1 State Key Laboratory of Eco-Hydraulics in Northwest Arid Region, Xi’an University of Technology, Xi’an 710048, China; szjkyzyyx@163.com (Z.S.); fly1836478971@163.com (L.F.); 2 Institute of Geotechnical Engineering, Xi’an University of Technology, Xi’an 710048, China; sjshao@xaut.edu.cn

**Keywords:** soil strength, failure criterion, spatial failure surface

## Abstract

In this paper, the common failure criteria of existing soil materials, such as the Mohr–Coulomb criterion, Drucker–Prager criterion, Lade–Duncan criterion, Matsuoka–Nakai criterion andAC-SMP criterion are systematically discussed, and the applicability of these criteria is quantitatively analyzed through the true triaxial test results of loess, so as to provide reference for the accurate selection of specific criteria in engineering practice. The failure criteria are classified from several aspects, such as whether the influence of the intermediate principal stress and the change of spatial moving plane are considered, analyzed and discussed, respectively. According to the true triaxial test results of undisturbed loess, the difference of strength criterion between the three-dimensional failure plane and p-q plane is analyzed, and based on the true triaxial test data of undisturbed loess, the error analysis of each failure criterion is carried out. The results show that the AC-SMP criterion is in good agreement with the test results, and can accurately evaluate the true triaxial test of loess. For different soil materials or different stress states, it is necessary to select appropriate failure criteria. This study shows how to choose the corresponding failure criterion under specific circumstances, so as to better satisfy the theory and practice and provide reference for engineering.

## 1. Introduction

Strength prediction of friction materials has always been the focus of engineering [1,2,3]. Many failure criteria have been proposed to describe the strength failure of various materials [4,5,6]. Human activities are closely related to rock and soil materials, and the failure strength of rock and soil materials is the core subject of rock and soil mechanics [7]. Common failure criteria include the Mohr–Coulomb criterion, Drucker–Prager criterion, Lade–Duncan criterion, Matsuoka–Nakai criterion, AC-SMP criterion and $\sqrt[{3}]{\sigma }-\mathrm{SMP}$ criterion.

The Mohr–Coulomb criterion (M-C) is one of the most widely used strength criteria in the geotechnical field [8]. The criterion considers the two-dimensional failure of materials. A large number of true triaxial tests has verified that the strength of geotechnical materials is closely related to the intermediate principal stress [9,10]. The Drucker–Prager criterion (D-P) considers the influence of hydrostatic pressure on yield and failure [11]. However, the strength envelope of this criterion is circular on the π plane, which has nothing to do with the stress Lode angle θ. This is inconsistent with the actual situation of soil. The Lade–Duncan criterion (L = D) [12] is an elastic-plastic model for sand proposed by Lade and Duncan in 1975. In 1977, they revised this model and changed the trajectories of the plastic potential plane, yield plane and failure plane on the p-q meridian plane to be curved, which reflected the influence of the confining pressure on the strength parameters of soil. The Matsuoka–Nakai criterion (SMP) [13] holds that the spatially mobilized plane is formed by the failure line composed of the Mohr–Coulomb criterion in any two principal planes. It can be considered that SMP criterion is the extension of the M-C criterion to three-dimensional space. It is widely used in the research field of soil strength because of considering the influence of the intermediate principal stress on soil strength. The axisymmetric compression spatially mobilized plane (AC-SMP) [14] holds that under the situation of $\sigma_{1}>\sigma_{2}=\sigma_{3}$, sliding failure may occur in either the $\sigma_{1}-\sigma_{2}$ plane or the $\sigma_{1}-\sigma_{3}$ plane. Considering the possible sliding failure in the above two principal stress planes, the $\sqrt[{3}]{\sigma }-\mathrm{SMP}$ criterion [15] is that $k\sqrt[{3}]{\sigma_{1}},k\sqrt[{3}]{\sigma_{2}}\;\mathrm{and}\;k\sqrt[{3}]{\sigma_{3}}$ determine the spatially mobilized plane of the intersection point of the large, intermediate and minimum principal stress axes of the soil element.

It is very important to choose a suitable geotechnical failure criterion for different soils [16]. For example, Khanna, K et al. conducted creep research by adopting the Tresca criterion for metal materials [17]; Singh, A et al. conducted vertical drilling research by adopting the Mohr–Coulomb criterion for rock materials [18], and Chen, HH et al. conducted anisotropy research by adopting the SMP criterion for soil materials [19]. However, the above scholars all adopted a single criterion to study the strength characteristics of a single material, but the quantitative analysis of applicability of these criteria to the real experimental results of the same soil material has never been involved. This paper compares and summarizes these commonly used failure criteria from different aspects, and makes an error analysis according to the true triaxial test of undisturbed loess, providing reference for theoretical research or engineering application of peer scholars.

## 2. Expression Forms of Several Strength Criteria

In the following strength criterion expressions, $\sigma_{1}$, $\sigma_{2}$ and $\sigma_{3}$, respectively, represent the maximum principal stress, intermediate principal stress and minimum principal stress, c is cohesion, φ is internal friction angle, $I_{1}$ and $I_{3}$, respectively, represent the first and third stress invariants, and $J_{2}$ is the second deviatoric stress invariant. The relationship between them and principal stress can be expressed by the following Equations (1)–(3) respectively:

(1) $$I_{1}=\sigma_{1}+\sigma_{2}+\sigma_{3}$$

(2) $$I_{3}=\sigma_{1}\sigma_{2}\sigma_{3}$$

(3) $$J_{2}=\frac{1}{6}\left[{\left(\sigma_{1}-\sigma_{2}\right)}^{2}+{\left(\sigma_{2}-\sigma_{3}\right)}^{2}+{\left(\sigma_{3}-\sigma_{1}\right)}^{2}\right]$$

### 2.1. Mohr–Coulomb Failure Criterion

The Mohr–Coulomb criterion (M-C), based on the results of the sand direct shear test, holds that the shear strength of soil can be expressed as a linear function of normal stress on a shear sliding surface. The Mohr–Coulomb strength criterion holds that when the stress ratio ${\left(\frac{\tau }{\sigma }\right)}_{\max}$ reaches the limit, the material will fail, and its expression is:

(4) $$\frac{\sigma_{1}-\sigma_{3}}{\sigma_{1}+\sigma_{3}+2c\cot\varphi }=\sin\varphi$$

### 2.2. Drucker–Prager Failure Criterion

The Drucker–Prager criterion (D-P) is based on the Mises criterion. Its expression is:

(5) $$\sqrt{J_{2}}-\alpha I_{1}-k=0$$

where α,k is the soil characteristic parameter.

### 2.3. Lade–Duncan Failure Criterion

The Lade–Duncan criterion (L-D) is a strength criterion based on the true triaxial test results of cohesionless sand, and its expression is:

(6) $$\frac{I_{1}^{3}}{I_{3}}=k_{f}$$

The revised Lade–Duncan criterion expression is:

(7) $$\left(\frac{I_{1}^{3}}{I_{3}}-27\right)*{\left(\frac{I_{1}}{p_{a}}\right)}^{m}-\eta_{1}=0$$

where $k_{f},\eta_{1}$ and m are material constants; the atmospheric pressure is 100 KPa.

### 2.4. Matsuoka–Nakai Failure Criterion

The Matsuoka–Nakai criterion (SMP) based on the Mohr–Coulomb criterion, the strength formula of soil under three-dimensional stress condition, is established. The criterion considers the influence of the intermediate principal stress on soil strength. The failure criterion holds that the soil is destroyed when the ratio of shear stress to normal stress reaches a certain value, and its expression is:

(8) $$\frac{2}{3}\sqrt{{\left(\frac{\sigma_{1}-\sigma_{2}}{2\sqrt{\sigma_{1}\sigma_{2}}}\right)}^{2}+{\left(\frac{\sigma_{2}-\sigma_{3}}{2\sqrt{\sigma_{2}\sigma_{3}}}\right)}^{2}+{\left(\frac{\sigma_{3}-\sigma_{1}}{2\sqrt{\sigma_{3}\sigma_{1}}}\right)}^{2}}=C$$

The SMP criterion given by Equation (8) is applicable to granular materials, that is, *C* = 0. For the case of *C* ≠ 0, it can be converted by the coordinate translation method, which is expressed as follows:

(9) $$\frac{2}{3}\sqrt{{\left(\frac{{\hat{\sigma }}_{1}-{\hat{\sigma }}_{2}}{2\sqrt{{\hat{\sigma }}_{1}{\hat{\sigma }}_{2}}}\right)}^{2}+{\left(\frac{{\hat{\sigma }}_{2}-{\hat{\sigma }}_{3}}{2\sqrt{{\hat{\sigma }}_{2}{\hat{\sigma }}_{3}}}\right)}^{2}+{\left(\frac{{\hat{\sigma }}_{3}-{\hat{\sigma }}_{1}}{2\sqrt{{\hat{\sigma }}_{3}{\hat{\sigma }}_{1}}}\right)}^{2}}=C$$

(10) $${\hat{\sigma }}_{i}=\sigma_{i}+\sigma_{0}$$

where *C* is the test constant.

### 2.5. AC-SMP Failure Criterion

The axisymmetric compression spatially mobilized plane failure criterion (AC-SMP) is based on the theory of the spatially mobilized plane. The spatially mobilized plane of soil under three-dimensional stress under axisymmetric compression was proposed, and its expression is:

(11) $$\frac{\tau_{N}}{\sigma_{N}}=\sqrt{\frac{\left(\sigma_{1}^{2}+\sigma_{2}^{2}K_{p}+\sigma_{3}^{2}K_{p}\right)\left(1+2K_{p}\right)}{{\left(\sigma_{1}+\sigma_{2}K_{p}+\sigma_{3}K_{p}\right)}^{2}}-1}=k_{f}$$

where $k_{f}$ is the test constant.

### 2.6. Failure Criterion

The $\sqrt[{3}]{\sigma }-\mathrm{SMP}$ criterion is based on the SMP criterion. According to the ratio of shear stress to normal stress on the sliding surface, the material is disrupted when the proportion reaches a certain value; its expression is:

(12) $$\frac{\tau_{N}}{\sigma_{N}}=\sqrt{\frac{(\sigma_{1}^{2}\left(\sigma_{2}^{\frac{2}{3}}+\sigma_{3}^{\frac{2}{3}}\right)+\sigma_{1}^{\frac{2}{3}}{\left(\sigma_{2}-\sigma_{3}\right)}^{2}+\sigma_{2}^{2}\sigma_{3}^{\frac{2}{3}}+\sigma_{2}^{\frac{2}{3}}\sigma_{3}^{2}-2\sigma_{1}\left(\sigma_{2}\sigma_{3}^{\frac{2}{3}}+\sigma_{2}^{\frac{2}{3}}\sigma_{3}\right)}{{\left(\sigma_{1}^{\frac{1}{3}}+\sigma_{2}{}^{\frac{1}{3}}+\sigma_{3}^{\frac{1}{3}}\right)}^{2}{\left(\sigma_{1}\sigma_{2}\sigma_{3}\right)}^{\frac{2}{3}}}}=k_{f}$$

## 3. Classification of Failure Criteria

### 3.1. According to Whether to Consider the Influence of the Intermediate Principal Stress

A large number of geotechnical tests have shown that the intermediate principal stress $\sigma_{2}$ has a significant influence on the strength of geotechnical materials, and the influence of the intermediate principal stress cannot be simply ignored in a practical application [20,21]. Based on different conditions, predecessors have put forward a variety of strength criteria considering the influence of $\sigma_{2}$. In geotechnical engineering, the M-C criterion has been widely used because of its clear concept and simple expression. Its shortcoming is that it does not consider the influence of the intermediate principal stress, which makes the calculation results conservative; the D-P criterion is developed on the basis of the Mises criterion. Although it considers the influence of the intermediate principal stress, it regards the influence of the intermediate principal stress and minimum principal stress on strength as the same, and the calculation results are overestimated. The trajectory of the L-D criterion in the π plane is a pear-shaped closed curve, which can basically reasonably reflect the influence of the intermediate principal stress on soil shear strength; based on the concept of the spatially mobilized plane, the SMP criterion considers that three Mohr circles have influence on soil strength, and considers the influence of the intermediate principal stress; the AC-SMP criterion holds that sliding failure may occur in the $\sigma_{1}-\sigma_{3}$ plane and the $\sigma_{1}-\sigma_{2}$ plane, and considering the possible sliding failure in the above two main planes, it also reflects the influence of the intermediate principal stress. The establishment of the criterion of AC-SMP and $\sqrt[{3}]{\sigma }-\mathrm{SMP}$ follows the research idea of the existing strength criterion, that is, it is established by the condition that shear stress and normal stress on a specific space surface (which can be collectively called the shear space sliding surface) when a variety of soils fail. Therefore, the shear stress and normal stress on these two kinds of space sliding failure surfaces can also reflect the influence of the intermediate principal stress. An interesting aspect of soil response is the sensitivity of mechanical behavior to intermediate principal stress [22]. If the influence of the intermediate principal stress is not taken into account, the calculated results will be inconsistent with the actual situation, which may cause intolerable catastrophic results when applied to engineering practice.

### 3.2. According to Whether the Spatial Plane Changes

The spatially mobilized plane of the Mohr–Coulomb criterion is parallel to the middle principal stress axis, and the cosine of the angle between its normal direction and the directions of the large, intermediate and minimum principal stresses are $\cos\left(45^{{}^{\circ}}+\frac{\varphi }{2}\right)$, 0 and $\cos\left(45^{{}^{\circ}}-\frac{\varphi }{2}\right)$, respectively, and the spatially mobilized plane is static. The spatially mobilized plane of the D-P criterion is the octahedral surface of the principal stress unit, and the cosine of the angle between the normal direction and the directions of the large, intermediate and minimum principal stresses is 33, so the normal direction is fixed and the spatial sliding surface is static. The spatially mobilized plane of the SMP criterion changes with the change of the failure stress state, and the cosine of the angle between the normal direction and the direction of the large, intermediate and minimum principal stresses are $\sqrt{I_{3}/\left(\sigma_{1}I_{2}\right)}$, $\sqrt{I_{3}/\left(\sigma_{2}I_{2}\right)}$ and $\sqrt{I_{3}/\left(\sigma_{3}I_{2}\right)}$, respectively. The spatially mobilized plane of the AC-SMP strength criterion does not change with the change of the failure stress state. The cosine of the angle between the normal direction and the direction of the large, intermediate and minimum principal stresses is $1/\sqrt{2k_{p}+1}$, $\sqrt{k_{p}}/\sqrt{2k_{p}+1}$, $\sqrt{k_{p}}/\sqrt{2k_{p}+1}$, respectively. The spatially mobilized plane is static. The criterion of the $\sqrt[{3}]{\sigma }-\mathrm{SMP}$ dynamic sliding surface changes with the change of the failure stress state, the direction cosine of the angle between the normal direction and the direction of the large, intermediate and minimum principal stresses are $\sqrt[{3}]{I_{3}}/\left(\sigma_{1}\sqrt{{\left(\sigma_{1}\sigma_{2}\right)}^{\frac{2}{3}}+{\left(\sigma_{2}\sigma_{3}\right)}^{\frac{2}{3}}+{\left(\sigma_{3}\sigma_{1}\right)}^{\frac{2}{3}}}\right)$, $\sqrt[{3}]{I_{3}}/\left(\sigma_{2}\sqrt{{\left(\sigma_{1}\sigma_{2}\right)}^{\frac{2}{3}}+{\left(\sigma_{2}\sigma_{3}\right)}^{\frac{2}{3}}+{\left(\sigma_{3}\sigma_{1}\right)}^{\frac{2}{3}}}\right)$, $\sqrt[{3}]{I_{3}}/\left(\sigma_{3}\sqrt{{\left(\sigma_{1}\sigma_{2}\right)}^{\frac{2}{3}}+{\left(\sigma_{2}\sigma_{3}\right)}^{\frac{2}{3}}+{\left(\sigma_{3}\sigma_{1}\right)}^{\frac{2}{3}}}\right)$, respectively. The spatially mobilized plane is dynamic. The directional cosine values of different strength criteria are shown in Table 1.

## 4. Comparative Analysis of Each Strength Criterion

### 4.1. Failure Surface Morphology

**Figure 1:**
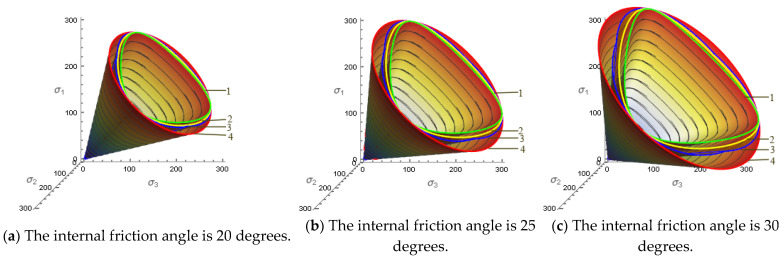
Three-dimensional failure surface morphology summary diagram of each strength criterion under different internal friction angles, 1—SMP criterion; 2—L-D criterion; 3—AC-SMP criterion; 4—D-P criterion. (**a**) The internal friction angle is 20 degrees; (**b**) The internal friction angle is 25 degrees; (**c**) The internal friction angle is 30 degrees.

**Figure 2:**
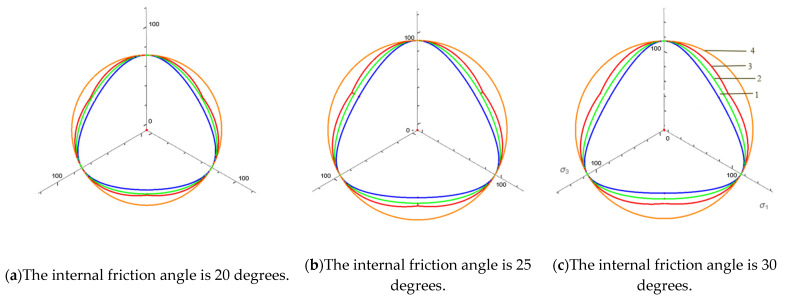
The failure envelope of each strength criterion in π plane under different internal friction angles, 1—SMP criterion; 2—L-D criterion; 3—AC-SMP criterion; 4—D-P criterion. (**a**) The internal friction angle is 20 degrees; (**b**) The internal friction angle is 25 degrees; (**c**) The internal friction angle is 30 degrees.

To visually compare the strength criteria of the M-C criterion, D-P criterion, L-D criterion, SMP criterion and AC-SMP criterion. According to the above Equations (4)–(8), and Equation (11), the shape of the space failure surface reflected in the principal stress space and the failure surface on the π plane are shown in Figure 1 and Figure 2.

According to the true triaxial test data of undisturbed loess, considering the stress space strength failure surface of soil with internal friction angles of 20 degrees, 25 degrees and 30 degrees. It can be seen from Figure 1, that the strength failure surface described by the L-D criterion and AC-SMP criterion is relatively close, which is between the D-P criterion and SMP criterion. With the increase in the internal friction angle, the opening of each criterion becomes larger.

In the following Figure 2, the envelope of each strength criterion with internal friction angles of 20 degrees, 25 degrees and 30 degrees was drawn on the π plane, considering that the failure lines of each criterion coincide under triaxial compression $\sigma_{1}>\sigma_{2}=\sigma_{3}$. The curve between b = 0 and b = 1 reflects the influence of the intermediate principal stress, where b is the ratio of the intermediate principal stress. In the triaxial elongation (b = 1), the SMP criterion corresponds to the lowest point of the failure limit line, while the D-P criterion corresponds to the highest point. This criterion is circular in the π plane and has nothing to do with the stress Lode angle θ, which is obviously inconsistent with the actual situation of soil. L-D and AC-SMP are between the above two criteria. The SMP criterion, L-D criterion and AC-SMP criterion all consider the difference between the triaxial compression and triaxial elongation strength. The failure surface of the AC-SMP criterion strength is composed of three sections, which, respectively, represent the three states of sliding surface in the principal stress unit. In Section 4.3, the error analysis of the strength failure of the four failure criteria was further carried out.

### 4.2. p-q Line

**Figure 3:**
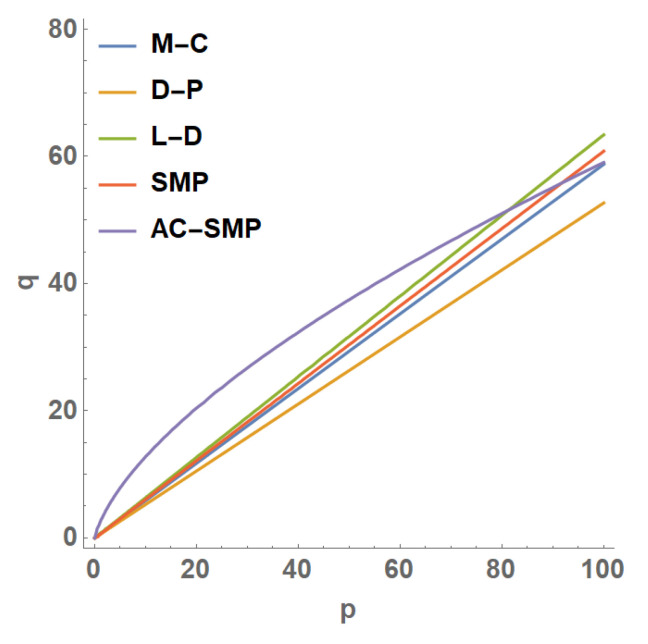
p-q line of each strength criterion.

The strength criterion of soil can be expressed in many different forms. For the convenience of analysis, the envelope of strength criterion is often drawn on the π plane and spatial meridian plane. Figure 2 was drawn on the envelope of the π plane. Considering sandy soil, c = 0 is reflected on the meridian plane according to the above Equations (4)–(8) and Equation (11). It can be seen from Figure 3 that except for AC-SMP being nonlinear, the p-q lines of other criteria are linear. When the spherical stress value was small, the eccentric stress value predicted by the AC-SMP criterion was higher, and with the gradual increase in the spherical stress value, the eccentric stress growth rate slowed down. The L-D criterion formed the upper envelope, the D-P criterion formed the lower envelope, and the M-C and SMP criterions were between D-P and L-D.

### 4.3. Verification and Comparison of True Triaxial Test Data

In order to further reflect the influence of each criterion on the intermediate principal stress, this paper quantitatively analyzed the stress range applicable to five strength criteria and the description of strength accuracy of soil under different intermediate principal stress ratio b stress paths by using the true triaxial test data of Xi’an Bailuyuan’s undisturbed loess.

The undisturbed loess used in this paper was taken from a slope scarp in Bailuyuan, the eastern suburb of Xi’an, Shanxi Province, China, and was naturally deposited. The soil sample was yellowish brown, with little calcareous nodules inside, belonging to silty clay loess. The natural water content was 17.0% and the dry density is 1.45 g/cm^3^. In the test, the cuboid undisturbed samples with specifications of 70 mm × 70 mm × 140 mm were used. In this test, the true triaxial tests under isotropic consolidation and drainage conditions were carried out, and the small principal stresses σ3 were controlled to be 50 kPa, 100 kPa, 150 kPa and 200 kPa, and the ratio coefficients of the medium principal stresses were 0, 0.25, 0.5, 0.75 and 1.0, respectively. At the beginning of the test, the isotropic consolidation stress was applied to the specimen through axial rigid and lateral flexible hydraulic blading at the same time. After consolidation, the ratio coefficients of the small principal stress and medium principal stress were controlled to remain unchanged in the shear stage, and the medium principal stress was applied. The loading deformation rate of the axial strain control mode was 0.05 mm/min until the specimen failed. The experimental results showed that the stress–strain relationship curve of undisturbed loess showed a hardening trend as a whole, and the peak value of the major stress difference was basically consistent with the volume deformation law, which was defined as the failure of the specimen with a strain of up to 12%, and the principal stress at failure was obtained. All the experiments in this study were carried out at room temperature (24–27 °C).

There are many evaluation methods for the error evaluation index [23,24]. The Levenberg–Marquardt optimization algorithm was adopted in this paper, and the evaluation index is shown in the following Equation (13):

(13) $$d=\sum_{i=1}^{N}|f((\sigma_{3})-\sigma_{1}^{test})|$$

where *d* is the sum of absolute values of fitting errors, $f(\sigma_{3})$ is $\sigma_{1}$ the value calculated according to the strength criterion, $\sigma_{1}{}^{test}$ is the $\sigma_{1}$ value of the true triaxial test, and *n* is the number of test data. The average relative error m was used to evaluate the accuracy of the strength criterion, and m was determined by the following Equation (14). The evaluation results are shown in Table 2:

(14) $$m=\frac{\sum_{i=1}^{N}\left|\frac{\left(f\left(\sigma_{3}\right)-\sigma_{1}^{test}\right)}{\sigma_{1}^{\mathrm{test}}}\right|}{N}\times 100\%$$

**Figure 4:**
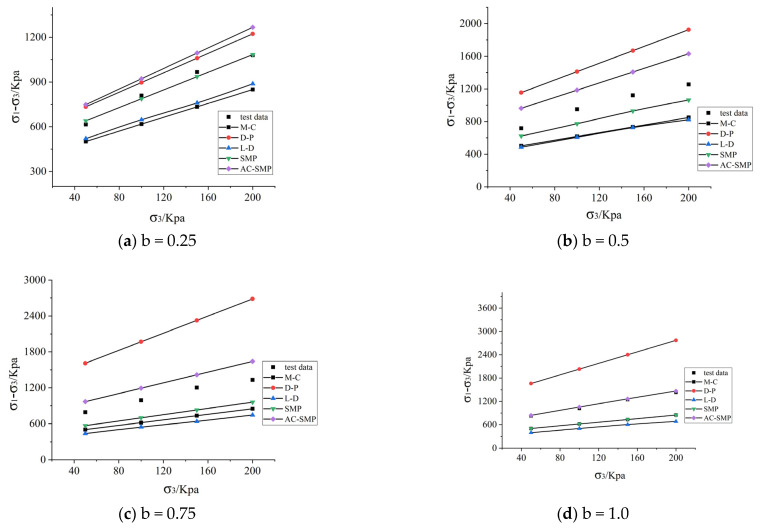
Comparison between true triaxial test data and strength criterion under different b values. (**a**) Comparison of true triaxial test data and strength criterion at b = 0.25. (**b**) Comparison of true triaxial test data and strength criterion at b = 0.5. (**c**) Comparison of true triaxial test data and strength criterion at b = 0.75. (**d**) Comparison of true triaxial test data and strength criterion at b = 1.

According to the fitting data of the conventional triaxial test (b = 0), the optimal parameters of each criterion were obtained. According to the optimal parameters, $\sigma_{2}$ and $\sigma_{3}$ were brought into each criterion, and $\sigma_{1}$ was calculated, which was compared with the test data and a line chart was created. Figure 4 shows the calculated failure strength under different intermediate principal stress ratios.

The M-C criterion does not consider the influence of the intermediate principal stress, so the failure strength will not vary with different b values, ignoring the influence of the intermediate principal stress, which leads to conservative results. The D-P criterion considers the influence of intermediate principal stress but considers the contribution of the intermediate and minimum principal stress to strength as equal, which leads to overvalued. Calculation results: It can be seen from Table 2 and Figure 4 below that only the relative error of the AC-SMP criterion increased first and then decreased with the increase in b value, and the prediction errors of the other three failure criteria for reflecting the change of the intermediate principal stress increased with the increase in b value. The average relative error of the AC-SMP criterion was 14%, which was the most accurate and obviously superior to other criteria for reflecting the change of intermediate principal stress in loess, followed by the SMP criterion with a relative error of 17%. The M-C criterion was applicable to conventional triaxial test data, but not to true triaxial test data.

### 4.4. Relationship of Strength Parameters

Different strength criteria have different strength parameters because of their different ideas. As the Mohr–Coulomb strength criterion has been widely used in ultimate strength, and engineers and technicians are familiar with the concepts and measurement methods of the c and φ values. If various failure lines coincide under triaxial compression and the failure plane passes through the origin, the relationship between various failure strength parameters and φ is as follows: if cohesion c ≠ 0, it can be obtained by coordinate translation. The finishing is shown in Table 3.

### 4.5. Relationship of Internal Friction Angle

On the basis of the M-C criterion, according to the D-P criterion, L-D criterion, SMP criterion, AC-SMP criterion and $\sqrt[{3}]{\sigma }-\mathrm{SMP}$ criterion, the relationship among these common criteria is established. In the following, only the detailed derivation process of the relationship between the compression internal friction angle and tension internal friction angle of the D-P criterion is given. The derivation process of other criteria is the same, and only the final result is given.

#### 4.5.1. Mohr–Coulomb Criterion

For cohesionless soil and c = 0, the above Equation (4) can be expressed as:

(15) $$\frac{\sigma_{1}-\sigma_{3}}{\sigma_{1}+\sigma_{3}}=\sin\varphi$$

Let $\frac{\sigma_{1}}{\sigma_{3}}=k_{p}$ and obtain:

(16) $$k_{p}=\tan^{2}\left(45^{{}^{\circ}}+\frac{\varphi }{2}\right)$$

#### 4.5.2. Drucker–Prager Criterion

Equation (5) can be expressed as:

(17) $$\frac{{\left(\sigma_{1}-\sigma_{2}\right)}^{2}+{\left(\sigma_{2}-\sigma_{3}\right)}^{2}+{\left(\sigma_{3}-\sigma_{1}\right)}^{2}}{{\left(\sigma_{1}+\sigma_{2}+\sigma_{3}\right)}^{2}}=k_{f}$$

Under the condition of axial symmetry, let $\sigma_{2}=\sigma_{3}$, and the above formula can be expressed as:

(18) $$\frac{2{\left(\sigma_{1}-\sigma_{3}\right)}^{2}}{{\left(\sigma_{1}+2\sigma_{3}\right)}^{2}}=k_{f}$$

According to the Mohr–Coulomb strength criterion, $\frac{\sigma_{1}}{\sigma_{3}}=k_{p}$, we obtain:

(19) $$k_{f}=\frac{2{\left(k_{p}-1\right)}^{2}}{{\left(k_{p}+2\right)}^{2}}$$

Therefore, the Drucker–Prager failure criterion can be expressed as:

(20) $$\frac{{\left(\sigma_{1}-\sigma_{2}\right)}^{2}+{\left(\sigma_{2}-\sigma_{3}\right)}^{2}+{\left(\sigma_{3}-\sigma_{1}\right)}^{2}}{{\left(\sigma_{1}+\sigma_{2}+\sigma_{3}\right)}^{2}}-\frac{2{\left(k_{p}-1\right)}^{2}}{{\left(k_{p}+2\right)}^{2}}=0$$

where $k_{p}=\tan^{2}(45^{{}^{\circ}}+\frac{\varphi }{2})$.

The axisymmetric extrusion condition $\sigma_{1}>\sigma_{2}=\sigma_{3}$, let $\eta =\frac{\sigma_{1}}{\sigma_{3}}=\frac{\sigma_{2}}{\sigma_{3}}=\tan^{2}\left(45^{{}^{\circ}}+\frac{\varphi_{AE}}{2}\right)$, the Drucker–Prager strength criterion can be expressed as:

(21) $$\frac{{\left(\sigma_{1}-\sigma_{2}\right)}^{2}+{\left(\sigma_{2}-\sigma_{3}\right)}^{2}+{\left(\sigma_{3}-\sigma_{1}\right)}^{2}}{{\left(\sigma_{1}+\sigma_{2}+\sigma_{3}\right)}^{2}}=\frac{2{\left(\eta -1\right)}^{2}}{{\left(2\eta +1\right)}^{2}}=\frac{2{\left(k_{p}-1\right)}^{2}}{{\left(k_{p}+2\right)}^{2}}$$

(22) $$\eta =\frac{\sqrt{A}+\sqrt{2}}{\sqrt{2}-2\sqrt{A}}$$

where $A=\frac{2{\left(k_{p}-1\right)}^{2}}{{\left(k_{p}+2\right)}^{2}}$.

#### 4.5.3. Lade–Duncan Criterion

(23) $$\eta =\frac{1}{24}(-12+A)-\frac{24A-A^{2}}{24{\left(216A-36A^{2}+A^{3}+24\sqrt{3}\sqrt{27A^{2}-A^{3}}\right)}^{\frac{1}{3}}}+\frac{1}{24}(216A-36A^{2}+A^{3}+{24\sqrt{3}\sqrt{27A^{2}-A^{3}})}^{\frac{1}{3}}$$

where $A=\frac{{\left(k_{p}+2\right)}^{3}}{k_{p}}$.

#### 4.5.4. Matsuoka–Nakai Criterion

(24) $$\eta =k_{p}$$

#### 4.5.5. AC-SMP Criterion

(25) $$\eta =\frac{\sqrt{A}k_{p}+1}{1-\sqrt{A}\left(k_{p}+1\right)}$$

where $A=\frac{2{\left(k_{p}-1\right)}^{2}}{9k_{p}{}^{2}\left(k_{p}+1\right)}$.

**Figure 5:**
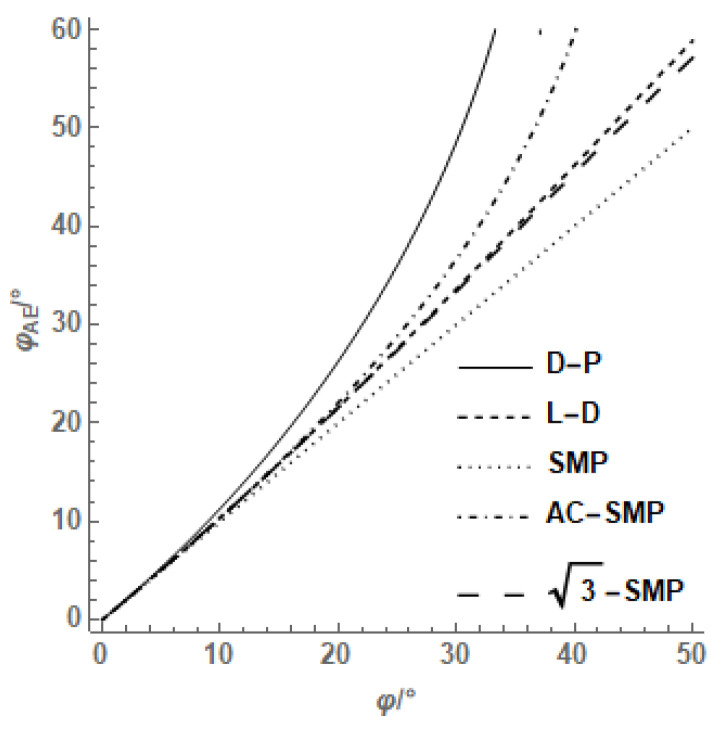
The relationship between φ and $\varphi_{AE}$.

According to the analysis of the above different criteria, the axisymmetric extrusion internal friction angle can be obtained from the axisymmetric compression internal friction angle, and the relationship between them is shown in Figure 5. The results show that the relationship between the extrusion internal friction angle and compression internal friction angle determined by the AC-SMP criterion was closer to the L-D criterion and SMP criterion, and was between the D-P criterion and L-D criterion. The $\sqrt[{3}]{\sigma }-\mathrm{SMP}$ strength criterion was approximately consistent with the L-D criterion.

The friction angles of SMP criterion under compression and extrusion conditions were consistent, but the results established by other criteria showed that the extrusion friction angle was larger than the compression friction angle. According to the M-C criterion of sand, the stress ratio of the triaxial compression failure in the π plane under axisymmetric condition:

(26) $$M_{c}=\frac{q_{c}}{p}=\frac{6\sin\varphi }{3-\sin\varphi }$$

Similarly, the stress ratio under triaxial elongation can be expressed as:

(27) $$M_{e}=\frac{q_{e}}{p}=\frac{6\sin\varphi_{AE}}{3+\sin\varphi_{AE}}$$

*R* is defined as the ratio of triaxial compression strength to the triaxial tensile strength of soil, as follows:

(28) $$R=\frac{M_{c}}{M_{e}}=\frac{\sin\varphi \left(3+\sin\varphi_{AE}\right)}{\sin\varphi_{AE}(3-\sin\varphi )}$$

**Figure 6:**
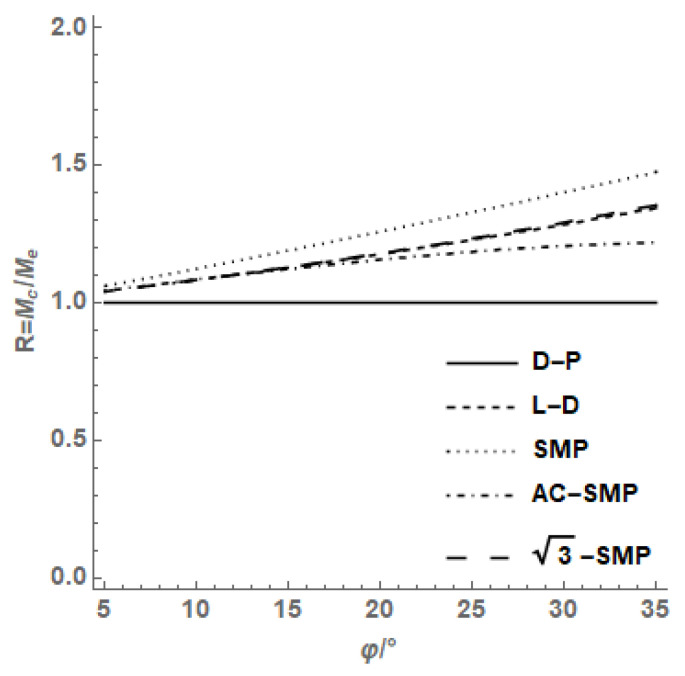
Changes of strength ratio with φ on symmetrical compression and extrusion.

According to the relationship between axisymmetric extrusion and the compression internal friction angle of different strength criteria shown in Figure 6, it can be obtained that the ratio of compression to extrusion strength of the AC-SMP criterion varies with the internal friction angle close to the L-D criterion, which is between the D-P criterion and SMP criterion, and the strength criterion is similar to the L-D criterion.

## 5. Discussion

Based on the above analysis and discussion, we can see that the Mohr–Coulomb failure criterion does not consider the influence of the intermediate principal stress, and its spatially mobilized plane was static, which belongs to a one kind of single shear strength theory. The failure plane of the Mohr–Coulomb failure criterion was orthogonal to the plane of the minimum principal stress and maximum principal stress, and the angle between it and the action plane of the maximum principal stress was 45°+φ2. The p-q line changed linearly. As it does not consider the intermediate principal stress, the strength error increased with the increase in b value, which was only suitable for describing conventional triaxial tests. The Drucker–Prager criterion considered the influence of the intermediate principal stress, and the normal direction was fixed, so the spatially mobilized plane was static, which belongs to the octahedral shear stress strength theory. As this criterion does not consider the influence of the Lode angle, the spatial sliding surface was a circle, and the p-q line also changed linearly. Although the D-P criterion considers the influence of the intermediate principal stress, it considers the contribution of the intermediate and minimum principal stress to strength as equal, which led to the calculation result being overestimated. The Lade–Duncan criterion, Matsuoka–Nakai criterion and $\sqrt[{3}]{\sigma }-\mathrm{SMP}$ criterion all consider the influence of the intermediate principal stress, and the spatially mobilized plane was dynamic, which belongs to the category of the octahedral shear stress strength theory. The spatial failure surfaces of the three criteria were also very similar, and the extrusion internal friction angle of the three criteria had little influence with the compression internal friction angle. Among them, the AC-SMP criterion was the best to describe the change of principal stress in loess, followed by the SMP criterion.

## 6. Conclusions

Based on the analysis of the common failure criteria, the common strength criteria were systematically compared and analyzed, and the following conclusions were drawn:

- The M-C criterion and D-P criterion are not suitable for describing the stress state of soil which is greatly influenced by the intermediate principal stress, such as soil with a large, buried depth and retaining structure of earth dam, etc. We should avoid using the M-C criterion and D-P criterion, and should use the AC-SMP and other failure criteria which consider the influence of the intermediate principal stress to meet the practical needs of engineering as much as possible.
- The sliding surfaces of the M-C criterion, D-P criterion and AC-SMP criterion are static, and the sliding surfaces of SMP criterion and $\sqrt[{3}]{\sigma }-\mathrm{SMP}$ criterion are dynamic, which provide a physical basis for studying the law of soil strength change. For soil media with vertical fissures and transversely isotropic sediments, its shear spatially mobilized plane must be related to soil structural characteristics. Considering the influence of soil structural anisotropy on the shear spatial sliding surface, studying the strength law of undisturbed soil can grasp its physical essence.
- Among the common failure criteria, only the relative error of the AC-SMP criterion first increases and then decreases with the increase in b value, and the prediction error of the other three failure criteria reflecting the change of the intermediate principal stress increases with the increase in b value. Among them, the AC-SMP criterion is the most accurate and superior to other criteria, followed by the SMP criterion, and the M-C criterion is suitable for conventional triaxial test data, but not for true triaxial test data. The AC-SMP and other criteria are preferred for friction materials with a strong structure.
- The internal friction angle of the D-P criterion changes the most with the axial symmetry compression, while the SMP criterion changes the least. The compression/extrusion strength ratio of the AC-SMP criterion is close to the L-D criterion, which is between the SMP criterion of the D-P criterion and strength criterion similar to the L-D criterion. The general strength criterion holds that the internal friction angle is a constant, but many studies have proved that it changes with the change of stress state, and correctly expressing the change of the internal friction angle can more accurately describe the failure of soil materials.

## Figures and Tables

**Table 1:** The direction cosine of different strength criterion.

	Normal Cosine	cosα	cosβ	cosγ
Criterion				
Mohr–Coulomb		$\cos\left(45^{{}^{\circ}}+\frac{\varphi }{2}\right)$	0	$\cos\left(45^{{}^{\circ}}-\frac{\varphi }{2}\right)$
Drucker–Prager		$\sqrt{3}/3$	$\sqrt{3}/3$	$\sqrt{3}/3$
Matsuoka–Nakai		$\sqrt{I_{3}/\left(\sigma_{1}I_{2}\right)}$	$\sqrt{I_{3}/\left(\sigma_{2}I_{2}\right)}$	$\sqrt{I_{3}/\left(\sigma_{3}I_{2}\right)}$
AC-SMP		$\frac{1}{\sqrt{2k_{p}+1}}$	$\frac{\sqrt{k_{p}}}{\sqrt{2k_{p}+1}}$	$\frac{\sqrt{k_{p}}}{\sqrt{2k_{p}+1}}$
$\;\;\;\sqrt[{3}]{\sigma }-\mathrm{SMP}$		$\frac{\sqrt[{3}]{I_{3}}}{\left(\sigma_{1}\sqrt{{\left(\sigma_{1}\sigma_{2}\right)}^{\frac{2}{3}}+{\left(\sigma_{2}\sigma_{3}\right)}^{\frac{2}{3}}+{\left(\sigma_{3}\sigma_{1}\right)}^{\frac{2}{3}}}\right)}$	$\frac{\sqrt[{3}]{I_{3}}}{\left(\sigma_{2}\sqrt{{\left(\sigma_{1}\sigma_{2}\right)}^{\frac{2}{3}}+{\left(\sigma_{2}\sigma_{3}\right)}^{\frac{2}{3}}+{\left(\sigma_{3}\sigma_{1}\right)}^{\frac{2}{3}}}\right)}$	$\frac{\sqrt[{3}]{I_{3}}}{\left(\sigma_{3}\sqrt{{\left(\sigma_{1}\sigma_{2}\right)}^{\frac{2}{3}}+{\left(\sigma_{2}\sigma_{3}\right)}^{\frac{2}{3}}+{\left(\sigma_{3}\sigma_{1}\right)}^{\frac{2}{3}}}\right)}$

**Table 2:** Fitting parameters and evaluation indexes of each strength criterion.

b Value Criterion		0	0.25	0.5	0.75	1.0
Mohr–Coulomb	c	106	106	106	106	106
	φ	33	33	33	33	33
	d	7.12	769	1342	1617	1813
	m	0.0024	0.22	0.33	0.37	0.40
Drucker–Prager	α	0.25	0.25	0.25	0.25	0.25
	k	125.7	125.7	125.7	125.7	125.7
	d	7.12	444	2118	4273	4348
	m	0.0024	0.13	0.53	0.99	0.97
Lade–Duncan	M_1_	1.4	1.4	1.4	1.4	1.4
	η_1_	1243.3	1243.3	1243.3	1243.3	1243.3
	d	7.12	655	1404	1955	2331
	m	0.0024	0.19	0.3	0.45	0.52
Matsuoka–Nakai	C	0.6	0.6	0.6	0.6	0.6
	σ_0_	166.6	166.6	166.6	166.6	166.6
	d	7.12	81	654	1265	1813
	m	0.0024	0.026	0.160	0.293	0.400
AC-SMP	k_f_	3.32	3.32	3.32	3.32	3.32
	σ_0_	166.6	166.6	166.6	166.6	166.6
	d	7.12	562	1141	901	114
	m	0.0024	0.17	0.29	0.21	0.025

**Table 3:** Strength parameter values of different strength criteria.

Criterion	Strength Parameters	
Mohr–Coulomb	*c*	*φ*
Drucker–Prager	$\alpha =\frac{2}{\sqrt{3}}\frac{\sin\varphi }{3-\sin\varphi }$	$k=2\sqrt{3}c\frac{\cos\varphi }{3-\sin\varphi }$
Lade–Duncan	$k_{1}=\frac{{\left(3-\sin\varphi \right)}^{3}}{1-\sin\varphi -\sin^{2}\varphi +\sin^{3}\varphi }$	-
Matsuoka–Nakai	$C=\frac{2\sqrt{2}}{3}\tan\varphi$	-
AC-SMP	kf=2[tan2(45°+φ2)−1]3tan32(45°+φ2)	-
$\sqrt[{3}]{\sigma }$-SMP	kf=2[tan2(45°+φ2)−1]tan32(45°+φ2)(tan32(45°+φ2)+2)	-

## Data Availability

The data underlying this article will be shared on reasonable request from the corresponding author.

## Abbreviations

The following abbreviations were used in this manuscript:

M-C	Mohr–Coulomb
D-P	Drucker–Prager
L-D	Lade–Duncan
SMP	Spatially mobilized plane
AC-SMP	Axisymmetric compression spatially mobilized plane
